# Towards a Food Pharmacy: Immunologic Modulation through Diet

**DOI:** 10.3390/nu11061239

**Published:** 2019-05-31

**Authors:** Ilse Molendijk, Sander van der Marel, P.W. Jeroen Maljaars

**Affiliations:** 1 Department of Gastroenterology and Hepatology, Leiden University Medical Center, 2313 ZA Leiden, The Netherlands; i.molendijk@lumc.nl; 2 Department of Gastroenterology and Hepatology, Haaglanden Medical Center, 2512 VA The Hague, The Netherlands; s.van.der.marel@haaglandenmc.nl

**Keywords:** inflammatory bowel disease, dietary modification, exclusive enteral nutrition, lifestyle modification, Mediterranean diet

## Abstract

Patients frequently wonder whether their dietary pattern influences the course of inflammatory bowel disease (IBD). Many patients even avoid certain foods that aggravate their symptoms. Although interest in nutritional interventions is rising among physicians, the current application of nutritional interventions in the IBD population is limited due to the lack of scientific evidence from clinical trials. Several studies, however, have identified associations between diet, gut microbiota, intestinal epithelial integrity, and mucosal immune responses. In patients consuming predominantly a Western diet high in n-6 poly-unsaturated fatty acids (PUFAs), sugars, and meat, and low in fruits and vegetables, an impaired gut epithelial barrier and disturbances in the intestinal microbiota have been observed, resulting in a chronic mucosal inflammation. An anti-inflammatory diet may restore this disbalance. In this review, we discuss the effects of diet on the composition of the microbiota, the gut epithelial barrier function, and the mucosal immune system.

## 1. Introduction

Inflammatory bowel diseases (IBDs), consisting of Crohn’s disease (CD) and ulcerative colitis (UC), are chronic immune-mediated disorders that can have a large impact on the wellbeing and quality of life of patients worldwide [1,2]. More than 1.5 million Americans are affected by IBDs, as are over 2.2 million Europeans [3], and the incidence of IBDs is increasing in newly industrialized countries in Africa, Asia, and South America [4]. Both diseases are characterized by a relapsing-remitting disease course, necessitating aggressive medical therapies in some cases [1,2]. 

Diet is important for patients with IBD. Patients often ask their physician whether the food they eat can influence the course of their inflammatory bowel disease. Very little data is available to answer this question. Data from studies in oncology show that eating or avoiding certain foods can increase or decrease the risk for developing colorectal cancer and other digestive cancers, and that this is probably related to the pro-inflammatory aspects of certain foods. This would suggest that nutrition may also affect both the pathogenesis and the disease course of IBD.

Additionally, it appears that dietary patterns in the decades before the onset of CD have been associated with the development of this disease. Patients who followed a healthy diet characterized by high fruit, vegetable, and fish intake during high school were 50% less likely to develop CD [5]. High intake of sugar and soft drinks combined with a low intake of vegetables was associated with UC risk [6].

These observations appear to be corroborated by observations in developing countries and among immigrants from developing countries: adaptation to a Western lifestyle with a Western diet increases both the incidence and prevalence of IBD in these populations [7,8]. The Western diet is high in fat (animal and milk fats, n-6 polyunsaturated fatty acids (PUFAs)) and refined sugars, and contains little fiber and healthy fats (n-3 PUFA). Apart from increasing the risk of developing IBD, the disease course may also be influenced by dietary patterns. Intake of a diet with a high fat content or a low ratio of pro-inflammatory n-6 to anti-inflammatory n-3 fats was associated with a higher level of disease activity [9,10,11].

This suggests that a Western diet is an environmental risk factor for developing IBD.

## 2. Diet, Symptoms, and Food Avoidance

A number of studies have assessed CD patients’ perception of the role of food and nutrients in gastrointestinal symptoms. Kinsey and Burden [12] found that 42% of IBD patients reported that food affected their symptoms “a lot” or “severely”, and 51% of the respondents indicated that diet was important or extremely important as a means of controlling symptoms. Another study found that an even higher percentage, around 70% of patients, thought that diet could trigger a disease flare [13]. Spicy foods are often considered a potential culprit: in a study by Limdi et al., 80% of the patients thought that eating food that was too spicy could result in a disease [14]. Specific items in the diet may aggravate symptoms such as abdominal pain, bloating, and diarrhea, whereas other dietary products alleviate these complaints. For instance, eating products with a high fat content, raw vegetables, and fruits and drinking carbonated beverages were perceived to increase the risk of a relapse by 40–50% of the patients [14]. The perceived relationship between specific foods and IBD complaints and disease activity leads to a high level of food avoidance. Casanova et al. [13] found that during remission, 77% of patients avoid certain foods, and this increased to 86% in patients with active disease. The avoidance of certain products increases the risk of vitamin and other biochemical deficiencies, and also increases the risk of malnutrition, which was estimated to occur in around 14% of the IBD population [13]. Currently, only a diet low in fermentable oligosaccharides, disaccharides, monosaccharides, and polyols (FODMAPS) has been shown to reduce complaints in a substantial part of the IBD population [15].

## 3. Exclusive Enteral Nutrition

Although certain dietary patterns may be related to a higher risk of developing IBD or a higher risk of disease activity, this does not show that dietary adjustments can influence the disease course or induce remission in patients with active disease. High quality studies in this area are lacking, and this is reflected in the results of a recent meta-analysis on the efficacy of the most studied dietary intervention, exclusive enteral nutrition (EEN), in which all conclusions were based on “very low quality evidence” [16]. EEN is a completely liquid formula containing all macro- and micronutrients a patient needs. EEN formulas can be classified as elemental (monomeric—free amino acids, glucose, oligosaccharides, and small amounts of essential fatty acids), semi-elemental (oligomeric—dipeptides, tripeptides, disaccharides, and medium chain triglycerides) or polymeric (whole protein, oligosaccharides, and long-chain triglycerides). Interventional studies have shown that clinical remission and mucosal healing can be achieved when a patient’s diet is replaced by exclusive enteral nutrition. However, the most successful studies have been performed in pediatric patients (Table 1). In a prospective trial, 34 children with newly diagnosed CD received EEN. Twenty-six patients completed at least 6 weeks of EEN. Early clinical remission at week 8 was achieved in 84% of the patients and 76% of the patients had early biochemical remission [17]. Early complete or nearly complete endoscopic mucosal healing was associated with a reduction in endoscopic relapse, anti-TNF (tumor necrosis factor) use, and hospitalization 1 year after EEN. In a 10-week prospective open label study on 37 children who had recently been diagnosed with active CD, 19 children received polymeric EEN to induce remission while 18 children received corticosteroids to induce remission. [18]. At week 10, clinical remission was comparable between both groups, but the number of patients with mucosal healing was significantly higher in the polymeric EEN group than the corticosteroid group (*p* < 0.05). A recent meta-analysis of studies in pediatric patients (consisting of the Borrelli [18] and Terrin [19] papers—see Table 1) found that 83% of pediatric patients achieved remission with EEN, compared to 61% on steroids, but this reached statistical significance only in the per-protocol analysis (Relative risk (RR) 1.43, 95% CI 1.03 to 1.97) [16]. EEN is currently the first-line treatment to induce remission in children with active CD [20,21]. No placebo-controlled trials have been performed in adults with active CD. However, in the controlled studies performed in adults comparing EEN with steroids, steroids appeared to be more effective in inducing remission than EEN [22,23,24], and a recent meta-analysis confirmed this: steroids were more effective in inducing remission than placebo (Table 1; RR 0.65, 95% CI 0.52 to 0.82; *p* < 0.05) [16]. There was no difference in efficacy between elemental, semi-elemental, and polymeric diets; however, a non-significant trend favoring very low fat and low long-chain triglycerides was observed. The lack of efficacy in adults may be due to non-compliance. Adults more often stop the EEN due to intolerance, and this may be related to the taste or to the method of delivery (a nasogastric tube) or unpalatability when the EEN is ingested orally [16]. Based on these studies, European Crohn and Colitis Organisation (ECCO) guidelines state that enteral therapy is only appropriate as an add-on treatment to support nutrition and not as a primary therapy in adults with active CD [25].

EEN has also been successful in preventing relapse in children with quiescent disease [29]. In adults, a prospective study randomly assigning 26 patients to a “half elemental” diet and 25 patients to a free diet resulted in a significantly lower relapse rate in the “half elemental” diet group after a mean follow-up of nearly 1 year compared to the patients who continued their own diet [26]. The compliance to the assigned intervention was similar in the two groups. In a study on 95 patients with CD in remission, 6-mercaptopurine (6-MP) was compared to an elemental diet and to a control group [27]. After 24 months, both the elemental diet and the 6-MP were significantly more effective in maintaining quiescent disease compared to patients in the control group (*p* < 0.05). No significant difference in the number of patients in remission was observed between the 6-MP and elemental diet group, suggesting that both interventions were equally able to maintain remission, although this may also be related to the relatively small sample size. A meta-analysis on EEN as an add-on treatment to anti-tumor necrosis factor (TNF) therapy found that the combination improved outcomes compared to anti-TNF without a dietary prescription [28]. Both the number of patients that reached remission as well as the number of patients that were in remission after a year increased significantly when the anti-TNF treatment was combined with the enteral nutrition (*p* < 0.01).

Together, this shows that enteral nutrition can induce remission, maintain remission, and improve the efficacy of other treatments. However, limited palatability and tolerability limit the applicability of this intervention.

## 4. Anti-Inflammatory Diet

Although EEN seems to reduce patients’ abdominal symptoms and inflammation [16,17,18], it is not completely clear how treatment with EEN results in these beneficial effects. However, in general, three mechanisms have been identified through which a diet could affect an inflamed bowel, although these mechanisms will undoubtedly interact. The current understanding of CD pathogenesis points to a continuum of interactions between the intestinal microbiota, the immune system, and the epithelial barrier function, and all three are amenable to specific environmental factors, such as diet [30,31]. We will discuss these three factors below.

## 5. Intestinal Barrier Function

Patients with active Crohn’s disease (CD) have an increased intestinal permeability. Even when little inflammation is present, intestinal integrity may still be reduced. This is associated with an elevated risk of a disease flare [32] and gastrointestinal complaints [33]. Intestinal permeability is controlled via tight junctions (TJs). TJs seal the paracellular space between the epithelial cells, thereby separating luminal content such as bacterial products and food antigens from the interstitium. TJs are selectively permeable for water, ions, and small molecules, however, a small proportion of luminal pathogens can cross the TJs. Patients that are susceptible to this translocated content develop an exaggerated mucosal immune response, resulting in an increased gut permeability—a “leaky gut” [34,35]. An increased intestinal permeability is also linked to the development of several diseases, including cardiovascular disease, diabetes, and non-alcoholic fatty liver disease (NAFLD) [35,36,37,38]. Improving intestinal barrier function may reduce gastrointestinal complaints and decrease the risk of a flare and the rise of metabolic diseases. The gut is continuously exposed to antigens in the food we consume. These dietary antigens may interact with the gut microbiota, changing its composition. This can result in an increased intestinal permeability, leading to an exaggerated immune response. Pathogens such as *Escherichia coli* O127:H6 (EPEC) and O157:H7 (EHEC), and bacterial toxins from, for instance, *Clostridium difficile* interfere with TJs between the epithelial cells, leading to an increased permeability. Also, the non-pathogenic *E. coli* strain C25 induces defects in the TJ complexes [39]. On the other hand, in a mouse model of necrotizing enterocolitis, *Bifidobacteria* preserved TJs and thereby intestinal barrier function [40]. The non-pathogenic yeast *Saccharomyces boulardii* supports barrier integrity and stimulates regeneration of damaged intestinal tissue in different diseases [41]. Dietary interventions such as EEN may be able to influence intestinal permeability [42,43]. In vitro, TNF-alpha decreased the TJ integrity of a monolayer culture. A polymeric formula completely restored the intestinal integrity of these monolayers [44]. Interleukin-10 knockout mice with colitis treated with EEN had an increased expression of TJs at a correct location and reduced epithelial apoptosis [45]. However, as discussed above, the lack of efficacy of EEN in adults may be due to the fact that EEN is unpalatable and/or that EEN is administered via a nasogastric tube. More palatable oral diets may be a solution for this problem. The Mediterranean diet (MD), rich in fresh vegetables, fruits, legumes, minimally processed whole-grains, nuts, fish, and olive oil, reduces systemic inflammation and is associated with increased diversity in gut microbiota and intestinal integrity [37,46,47].

## 6. Gut Microbiota

Our intestines are colonized with large numbers of bacteria, archaea, viruses, and eukaryotic microbes that form the gut microbiota [48]. The microbiota protects us against pathogens, regulates local immune responses, and has an important impact on host metabolic functions. In the first three years of life, the microbiota is shaped [49]. Next to antibiotic therapy and severe illness in these years, diet influences the composition of the microbiota. The composition of the microbiota depends on our dietary patterns, which can have both short-term and long-term effects on the variety of the gut microbiota [50]. There is a wide variation in the gut microbiota composition in the elderly [51]. These variations are associated with specific food patterns of these people and separate healthy elderly from those in long-term residential care [50]. The microbiota of the latter has been shown to be significantly less diverse than that of healthy elderly, resulting in increased frailty [50]. Changes in the diversity of the microbiota have been correlated with an increased incidence of chronic inflammatory diseases such as obesity [52], type 2 diabetes [53], cardiovascular disease [54,55], asthma [56], rheumatoid arthritis [57], colorectal cancer [58], and IBD [59,60].

There is growing evidence that a predominantly Western diet, high in fat and refined sugars, influences the composition of the microbiota. Consumption of high-fat milk and sugar-sweetened soda is linked to a lower diversity, whereas buttermilk and high polyphenol content beverages are associated with high microbiota diversity [61]. De Filippo et al., compared the gut microbiota of children aged 1–6 years living in Florence (Italy), where they consume a typical diet of the developed world, with children in Boulpon, a village in Burkina Faso, where they eat a traditional rural African diet high in fibers [62]. The microbiota of the children from Boulpon comprised less Firmicutes and Enterobacteriaceae (*Shigella* and *Escherichia*) compared to the children from Florence. *Prevotella*, *Xylanibacter* (Bacteroidetes), and *Treponema* (Spirochaetes) were exclusively present in the microbiota of the children from Boulpon, possibly as a result of high fiber intake. These bacteria can ferment xylan and cellulose, resulting in the production of short-chain fatty acids (SCFAs). SCFAs are an important source of energy for the mucosa, regulate homeostasis in the colon, and maintain an anti-inflammatory state [63]. Marchesi et al. [64] demonstrated that CD patients have decreased levels of butyrate, an abundant bioactive SCFA, and reduced levels of SCFAs in general in comparison to healthy subjects. A plant-based diet rich in polysaccharides and low in fat and sugars may select for SCFAs-producing bacteria and may be used in the maintenance of remission in patients with inflammatory diseases such as IBD. Microbial structures can rapidly be altered by a change in diet. In a two-week food exchange trial, African Americans consumed a high-fiber/low-fat African diet and rural Africans consumed a high-fat/low-fiber Western diet [65]. “Africanization” of the diet led to a 2.5-fold increase of butyrate in the colon as a result of fermentation of fibers by changes in microbiota, whereas in Africans consuming a Western diet, the amounts of butyrate were reduced by 50%. In concordance, a study among 14 obese men showed that diet-driven changes occurred within 3–4 days but were also reversed within the same time frame [66]. 

## 7. Immune Responses

The consumption of processed foods in developed countries has led to an excess of salt intake. Increased dietary salt intake is a risk factor for the development of cardiovascular diseases and autoimmune diseases through the induction of pathogenic T helper 17 (Th17) cells [67], a cell type which plays a pivotal role in the pathogenesis of CD [30]. Mutations in the nucleotide-binding oligomerization domain-containing protein 2 (NOD2) gene is associated with CD. NOD-like receptors (NLRs) sense muramyl dipeptide (MDP), present in all Gram-negative and -positive bacteria, leading to bacterial clearance. Mutations in the NOD2 gene, as seen in patients with IBD, are linked to less clearance of bacteria and suppression of the anti-inflammatory cytokine interleukin (IL)-10 [68]. Another family of innate immune receptors associated with IBD are the Toll-like receptors (TLRs). TLRs are expressed by dendritic cells, and TLR2 and TLR4 are upregulated under inflammatory conditions [2]. TLR2 and TLR4 are increased in the mucosa of patients with IBD compared to healthy controls, resulting in the loss of mucosal homeostasis [68]. Specific dietary patterns can influence the expression of both families of innate immune receptors. For instance, a diet high in fat and carbohydrates increased the expression of TLR2 and TLR4 [69]. Plasma concentrations of lipopolysaccharide (LPS) were also increased [69]. The consumption of orange juice during this meal, however, prevented the enhanced expression of both TLRs and LPS. This was probably due to the reactive oxygen species (ROS)-suppressive effect of flavonoids (naringenin and hesperidin) in the orange juice. A meal high in fibers did not result in an increased concentration of LPS and the induction of TLR2 and TLR4. The authors concluded that, although the content of LPS in a high-fiber meal is comparable to a meal high in fat and carbohydrates, the inflammatory nature of the latter possibly reduced the integrity of the mucosal barrier that normally prevents the translocation of bacterial products from the gut. In a mouse model of CD, a Western diet induced gene transcription of NOD2 and increased gut permeability, allowing the adherent-invasive *E. coli* to colonize, which resulted in an inflammatory state [70]. Together, these data suggest that a Western diet, high in salt, fat, and carbohydrates, may contribute to the development of IBD in predisposed patients, and to the loss of remission in patients with quiescent disease.

## 8. A Mediterranean Diet as an Anti-Inflammatory Diet

A Mediterranean diet (MD) is able to promote a healthy microbiota, reduce systemic inflammation, and reduce intestinal permeability. Two clinical trials evaluating the effect of a MD have been performed in patients with rheumatoid arthritis (RA) and were systematically reviewed by Forsyth et al. [71]. McKellar et al. [72] assigned patients with RA aged 30–70 years to either a 6-week hands-on cooking workshop (2 h per week delivered by nutritionists and teaching staff from local colleges), whereas the control group received a hand out with the general principles of a healthy diet. In this study, the MD reduced symptoms and improved physical functioning. The second study was a randomized controlled trial by Sköldstam et al. [73]. In this study, patients were randomized between a Cretan diet versus an ordinary Western diet. Symptoms, physical functioning, and biomarkers of inflammation (i.e., C-reactive protein (CRP)) improved in the intervention arm. The authors of the systematic review concluded that joint pain could be reduced and joint function could be improved using a MD. In addition, the risk of cardiovascular disease, which is substantially elevated in RA patients, could also be reduced by a MD. Our group recently performed a trial in 38 IBD patients using a group-based Mediterranean diet intervention (Molendijk et al., ECCO 2019) [74]. Patients with quiescent IBD that were willing to change their lifestyle, including dietary habits, were enrolled. Support was provided during three instruction and feedback days, and patients were able to use an online peer support platform. In this study, we found that 70% of patients were able to alter their dietary habits toward a more Mediterranean pattern. Second, we found that quality of life, as assessed with the short-IBD questionnaire, was increased and CRP was decreased in the active group. The magnitude of the changes in CRP and quality of life was correlated to the magnitude of the dietary change.

Together, these data suggest that a MD can increase the wellbeing of patients with inflammatory disorders. Whether a MD can alter the disease course or reduce inflammation is uncertain. However, the fact that the CRP was reduced in the Mediterranean arm is in this respect encouraging.

## 9. Conclusions

Recent studies have shown promising results of dietary alterations, such as the introduction of a MD, on the three most important players in the pathogenesis of IBD: intestinal permeability, gut microbiota composition, and inflammatory responses. Although patients are interested in lifestyle advice and a subset of physicians is eager to employ lifestyle-related interventions, the current application of lifestyle interventions in clinical practice is limited. This has a number of causes, such as lack of evidence-based interventions (which intervention works) and evidence-based practices (how to apply the intervention in clinical practice). What is clear is that networks have to be established, with physicians of different backgrounds, dieticians, physical therapists, and (physician-)scientists as participants. These collaborations will facilitate the development and implementation of these interventions.

## Figures and Tables

**Table 1:** Details of papers on EEN referenced in the manuscript.

Reference	Population	Intervention	Control	Duration	Outcome
**Induction of remission**					
Narula [16]	Meta-analysis of six studies, 352 adults	Enteral nutrition (EN)	Corticosteroids (CS)		Clinical remission EN: 45% vs. CS: 73%, *p* < 0.05
Grover [17]	34 children	Nutrison	No control	8 weeks	Clinical remission 84%; mucosal healing 42%
Borrelli [18]	37 children	Polymeric diet (PD)	Corticosteroids (CS)	10 weeks	Clinical remission PD: 79% vs. CS: 67%, *p* = ns. Mucosal healing PD: 74% vs. CS: 33%, *p* < 0.05
Terrin [19]	20 children	Semi-elemental diet (SED)	Corticosteroids (CS)	8 weeks	Clinical remission SED: 90% vs. CS: 50%, *p* < 0.01
**Maintenance of remission**					
Takagi [26]	51 adults	Half-elemental diet (HED)	Regular Diet (RD)	1 year	Relapse rate 1 year 35% in HED vs. 64% in RD
Hanai [27]	95 adults	Elemental diet (ED)	6-Mercaptopurin (6-MP), no intervention	2 years	Relapse rate: ED 53%, 6-MP 40%, control 73% (*p* < 0.05 ED vs. control; 6-MP vs. control)
**EEN as co-medication**					
Nguyen [28]	Meta-analysis of four studies, 342 adults	Specialized enteral nutrition combined with infliximab (combo)	Infliximab monotherapy	1 year	Induction: 69.4 in combo vs. 45.4 in mono (*p* < 0.01); Maintenance: Combo 74.5%, mono 49.4% (*p* <0.01)

CD: Crohn’s disease; EEN: exclusive enteral nutrition. For a full review of the efficacy of EEN in inducing remission in active Crohn’s disease, please see Narula et al. [16].
